# *In Vitro* Activity of Rifabutin and Rifampin against Antibiotic-Resistant Acinetobacter baumannii, Escherichia coli, Staphylococcus aureus, Pseudomonas aeruginosa, and Klebsiella pneumoniae

**DOI:** 10.1128/msphere.00920-21

**Published:** 2021-11-24

**Authors:** Bosul Lee, Jun Yan, Amber Ulhaq, Sarah Miller, Wonjae Seo, Peggy Lu, Rosemary She, Brad Spellberg, Brian Luna

**Affiliations:** a Department of Molecular Microbiology and Immunology, Keck School of Medicine at USC, Los Angeles, California, USA; b Department of Pathology, Keck School of Medicine at USC, Los Angeles, California, USA; c Los Angeles County-USC Medical Center, Los Angeles, California, USA; Antimicrobial Development Specialists, LLC

**Keywords:** *A. baumannii*, *E. coli*, *Klebsiella*, *Pseudomonas aeruginosa*, *S. aureus*, rifabutin, rifampin

## Abstract

We recently reported that the antimicrobial activity of rifabutin against Acinetobacter baumannii is best modeled by the use of RPMI for *in vitro* susceptibility testing. Here, we define the effects of medium on the susceptibility and frequency of resistance emergence in a panel of A. baumannii, Escherichia coli, Staphylococcus aureus, Klebsiella pneumoniae, and Pseudomonas aeruginosa clinical isolates. Only A. baumannii was hypersusceptible to rifabutin *in vitro* and *in vivo* using a Galleria mellonella infection model. *In vitro*, the frequency of resistance emergence was greater when the bacteria were selected on RPMI versus tryptic soy agar (TSA) or Mueller-Hinton II (MHII) agar plates. However, the frequency of resistance emergence was lower *in vivo* than in the RPMI *in vitro* condition.

**IMPORTANCE** Rifabutin has been recently described as a potential adjunctive therapy for antibiotic-resistant A. baumannii infections due to hypersensitivity in iron-depleted media, which may more closely mimic an *in vivo* environment. Here, we report that this hyperactivity is specific for A. baumannii, rather than being a general effect for other pathogens.

## INTRODUCTION

The increasing emergence of antibiotic-resistant bacteria is causing a global health crisis (1). In the United States, more than 2.8 million people are infected with antibiotic-resistant bacteria annually, which results in 35,000 deaths per year (2). Extensively multidrug-resistant Acinetobacter baumannii, specifically, is responsible for approximately 23,000 and 75,000 cases of infections annually in the U.S. and globally (in developed countries), resulting in approximately 10,000 and 30,000 deaths per year, respectively (3). As such, the U.S. Centers for Disease Control and Prevention (CDC) have labeled A. baumannii as an urgent threat due to lack of available treatment options (2).

Standard MIC testing utilizes the nutrient-rich medium Mueller-Hinton II (MHII) for evaluating the *in vitro* activity of antibiotics (4, 5). However, the reliance on MHII media may be problematic due to their lower accuracy for predicting *in vivo* antimicrobial efficacy for some drugs (6–8). For example, we showed recently that rifabutin (RFB) possesses hyperactivity against A. baumannii in RPMI medium (MIC < 0.05 mg/L) but not in rich MHII medium (9, 10). Under the low-iron and low-amino-acid conditions of RPMI (but not in MHII), A. baumannii *fhuE* is upregulated and rifabutin is able to hijack this transporter to rapidly enter the bacteria (9, 11). The *in vitro* hyperactivity of rifabutin in RPMI, rather than in MHII medium, predicted *in vivo* efficacy (9, 10). In this study, we used more than 100 A. baumannii clinical isolates in MIC assays to define the robustness of the activity across a broader array of strains.

Untested in previous studies were the *in vitro* and *in vivo* activities of rifabutin and rifampin (RIF) against other Gram-positive and -negative bacteria, as well as the impact of the culture medium on the emergence of resistance to rifabutin. In the current study, we determined if other bacteria are also more susceptible to rifabutin under low-iron conditions by quantifying rifabutin and rifampin MICs against a panel of multidrug resistance (MDR) A. baumannii strains, extended-spectrum-beta-lactamase (ESBL)-producing Escherichia coli, methicillin-resistant Staphylococcus aureus (MRSA), carbapenem-resistant (CR) Klebsiella pneumoniae, and MDR Pseudomonas aeruginosa clinical isolates to determine if other bacteria are also observed to be more susceptible to rifabutin when the MIC assay is conducted under low-iron conditions. We also evaluated the efficacy of rifabutin and rifampin *in vivo* against representative isolates of the aforementioned bacterial species using a Galleria mellonella infection model. Finally, we sought to determine if the hyperactivity of rifabutin but not rifampin against A. baumannii altered the frequency of emergence of drug-resistant mutants *in vitro* and *in vivo*.

## RESULTS

### MIC distributions.

**FIG 1:**
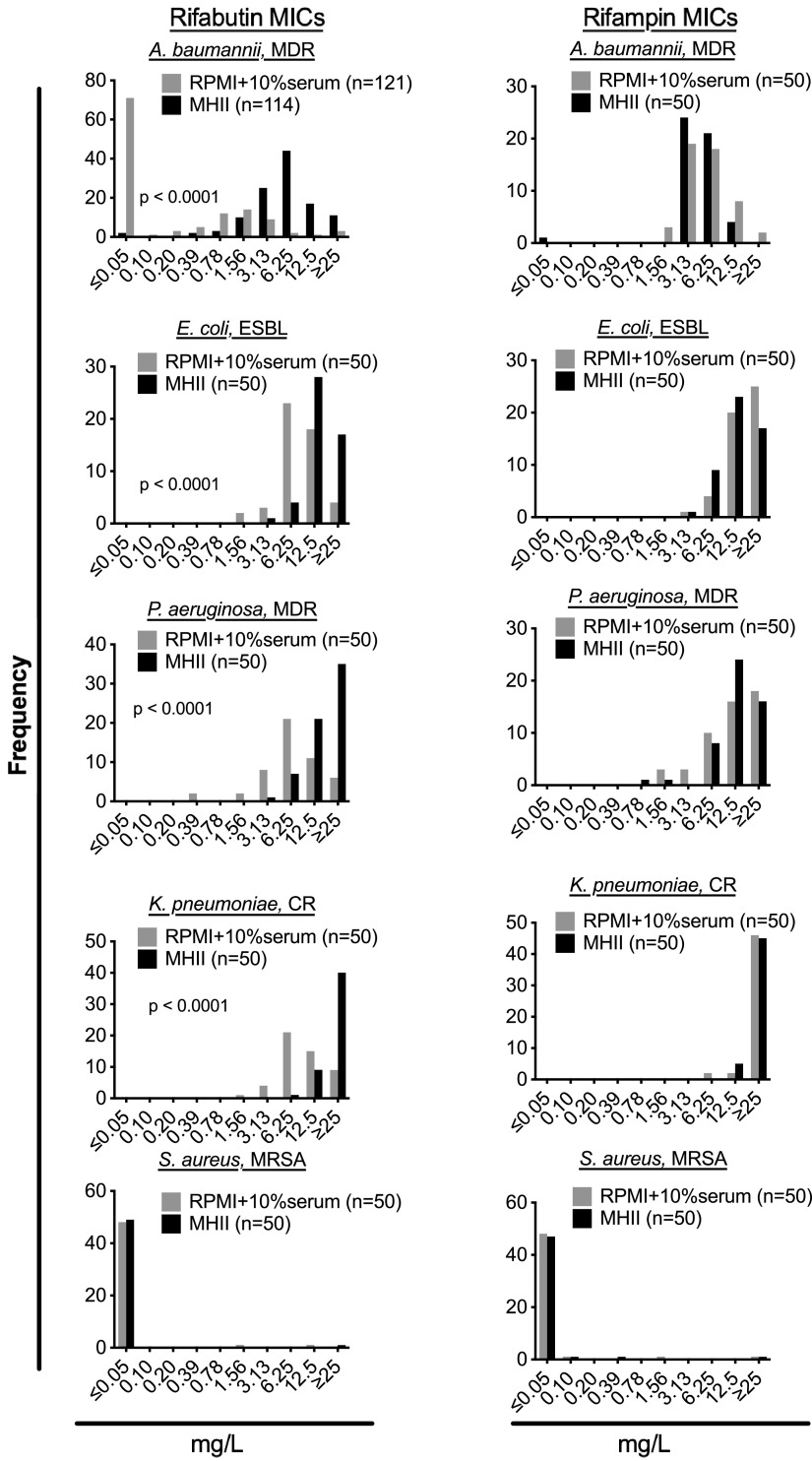
Distribution of rifabutin and rifampin MICs. MICs for both rifampin and rifabutin were determined using either standard MHII culture conditions or modified RPMI plus serum culture conditions for A. baumannii, S. aureus, E. coli, K. pneumoniae, and P. aeruginosa. The numbers of clinical isolates tested are given in the symbol keys. Statistical comparisons were made by Mann-Whitney test comparing MICs obtained in RPMI and MHII.

**FIG 2:**
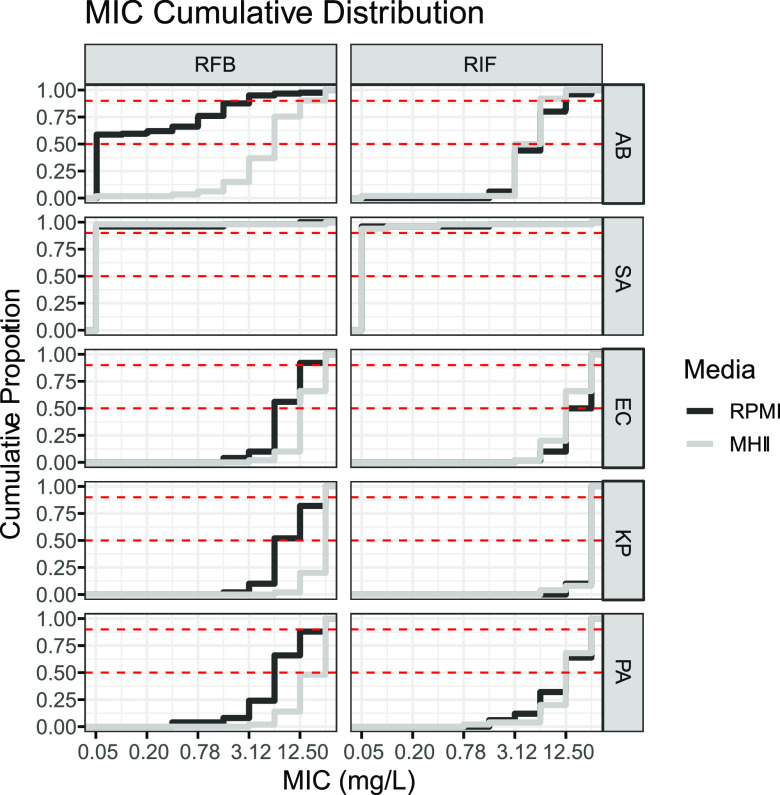
Cumulative distribution of MICs. Red dashed lines indicate the MIC_50_ and MIC_90_ cutoffs. AB, A. baumannii; SA, S. aureus; EC, E. coli; KP, K. pneumoniae; PA, P. aeruginosa.

Significant shifts in the distribution of rifabutin MICs in MHII versus RPMI were observed for A. baumannii, E. coli, P. aeruginosa, K. pneumoniae, and S. aureus clinical isolates (Mann-Whitney test, *P* < 0.0001) (Table 1; Fig. 1 and 2). However, the shift in MIC was greatest for A. baumannii isolates, as evidenced by a >125-fold and 8-fold shift in the rifabutin MIC_50_ and MIC_90_, respectively (Table 1; Fig. 1 and 2). In contrast, small rifabutin MIC_50_ shifts were observed for E. coli (2-fold), P. aeruginosa (2-fold), and K. pneumoniae (4-fold) (Table 1; Fig. 1 and 2). No significant shift in rifabutin or rifampin MICs for S. aureus isolates was observed when values obtained in MHII were compared to those obtained in RPMI broth (Table 1; Fig. 1 and 2).

**TABLE 1:** Summary of MIC_50_s and MIC_90_s

Bacterium	*n*	Drug	Medium	MIC (mg/L)	
				50%	90%
A. baumannii	114	RFB	MHII	6.25	12.5
	121	RFB	RPMI	≤0.05	1.56
	50	RIF	MHII	3.13	6.25
	50	RIF	RPMI	6.25	12.5
E. coli	50	RFB	MHII	12.5	≥25
	50	RFB	RPMI	6.25	12.5
	50	RIF	MHII	12.5	≥25
	50	RIF	RPMI	12.5	≥25
S. aureus	50	RFB	MHII	≤0.05	≤0.05
	50	RFB	RPMI	≤0.05	≤0.05
	50	RIF	MHII	≤0.05	≤0.05
	50	RIF	RPMI	≤0.05	≤0.05
P. aeruginosa	50	RFB	MHII	12.5	25
	50	RFB	RPMI	6.25	25
	50	RIF	MHII	12.5	25
	50	RIF	RPMI	12.5	25
K. pneumoniae	50	RFB	MHII	25	25
	50	RFB	RPMI	6.25	25
	50	RIF	MHII	25	25
	50	RIF	RPMI	25	25

### *In vivo* rifampin and rifabutin efficacy.

**FIG 3:**
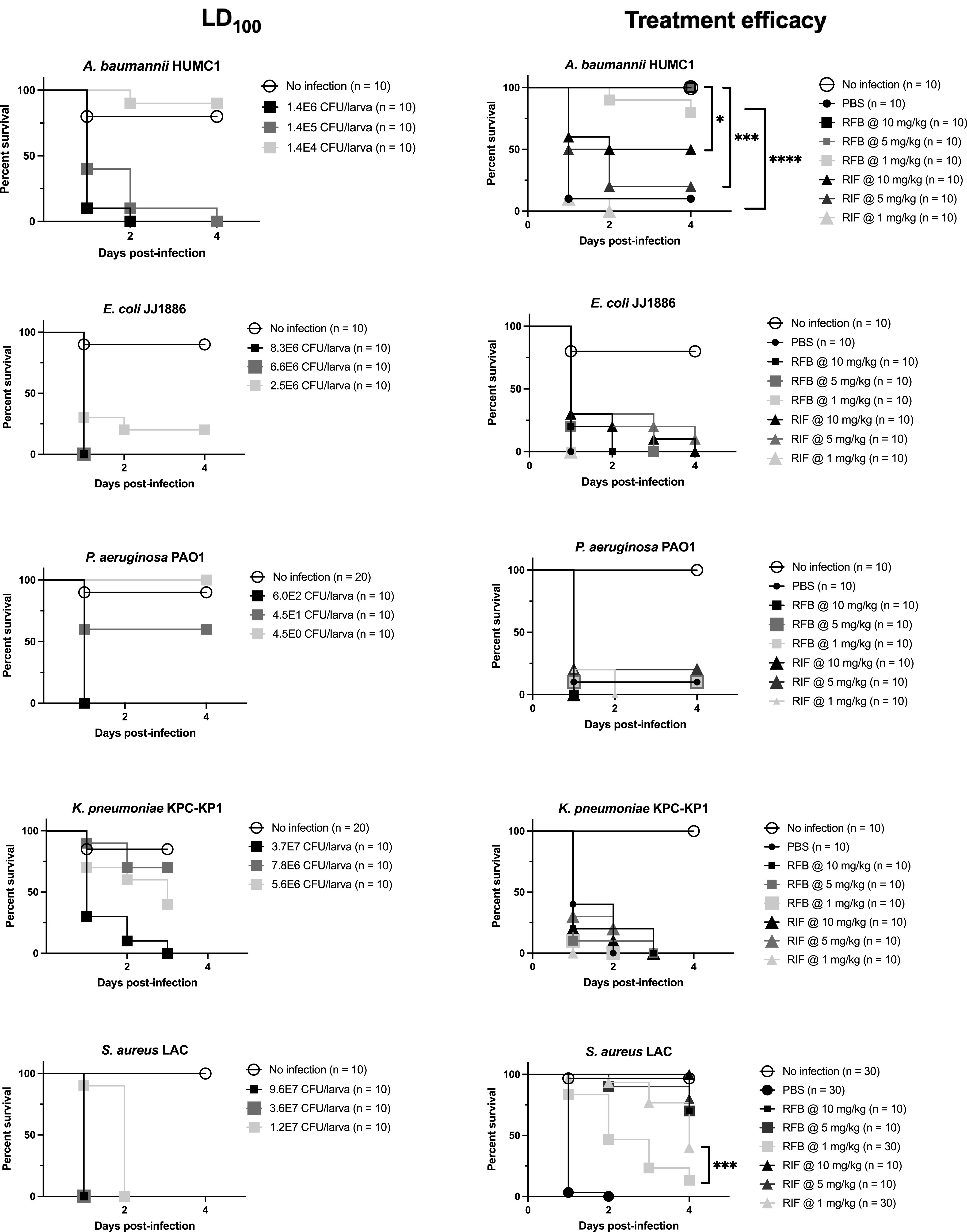
*In vivo* rifampin and rifabutin efficacy. Larvae were challenged with various bacterial inocula to determine each strain’s LD_100_. No-infection controls received PBS. To determine treatment efficacy, larvae were challenged with 1.1E6 CFU/larva A. baumannii HUMC1, 7.6E6 CFU/larva E. coli JJ1886, 5.5E2 CFU/larva P. aeruginosa PAO1, 3.4E7 CFU/larva K. pneumoniae KPC-KP1, or 2.2E7, 3.3E7, or 2.0E7 CFU/larva S. aureus LAC before being treated with PBS, RFB, or RIF. No-infection controls received 2 doses of PBS. Statistical comparisons, which are not significant unless indicated, were made by a log rank (Mantel-Cox) test comparing survival between dose-equivalent drug-treated groups.

*In vivo* survival of Galleria mellonella challenged with A. baumannii, E. coli, P. aeruginosa, K. pneumoniae, or S. aureus and treated with rifampin or rifabutin was consistent with RPMI MIC testing results (Fig. 3; Table 2). Specifically, rifabutin was more effective than rifampin at equivalent doses to rescue *Galleria* from A. baumannii infection (log rank test: *P* = 0.011 at 10 mg/kg; *P* = 0.0003 at 5 mg/kg; *P* < 0.0001 at 1 mg/kg) (Fig. 3). There was no significant difference between rifabutin’s and rifampin’s abilities at all tested doses to rescue *Galleria* infected with E. coli, P. aeruginosa, or K. pneumoniae. Though there was no significant difference between rifabutin’s and rifampin’s efficacies at 10 mg/kg or 5 mg/kg against S. aureus infection, rifampin was better able than rifabutin at 1 mg/kg (log rank test, *P* = 0.0001) to rescue *Galleria* (Fig. 3).

**TABLE 2:** MICs of strains used in Galleria mellonella infections

Strain	Medium	MIC (mg/L) of:	
		RFB	RIF
A. baumannii HUMC1	MHII	3.13	3.13
	RPMI	≤0.05	25
E. coli JJ1886	MHII	6.25	3.13
	RPMI	6.25	25
P. aeruginosa PAO1	MHII	25	6.25
	RPMI	25	25
K. pneumoniae KPC-KP1	MHII	25	25
	RPMI	12.5	>25
S. aureus LAC	MHII	≤0.05	≤0.05
	RPMI	≤0.05	≤0.05

### *In vitro* rifampin and rifabutin mutation frequency.

**FIG 4:**
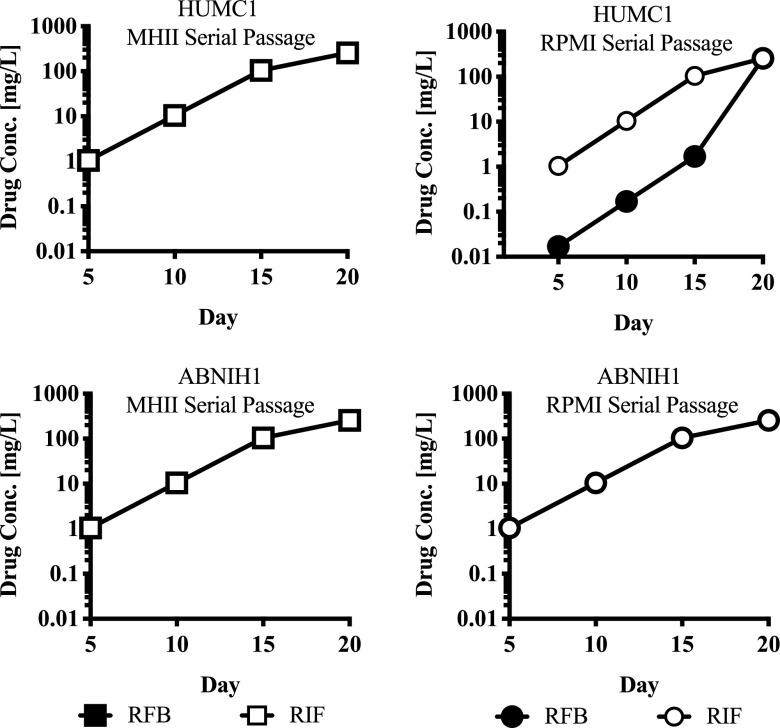
20-day serial passage mutant selection. For HUMC1, the starting MIC for both drugs was 3.13 mg/L in MHII. In RPMI, however, the starting MICs for rifampin and rifabutin were 3.13 mg/L and <0.05 mg/L, respectively. For ABNIH1, the starting MIC for both drugs was 3.13 mg/L in both MHII and RPMI. Despite HUMC1 initially being more susceptible to rifabutin in RPMI, it was still possible to select high-level resistance mutants. Similarly, no difference in rifabutin resistance emergence was observed between the hypersusceptible strain HUMC1 and the susceptible strain ABNIH1 after serial passage.

We expanded on previous efforts to study mutation frequency (9) by comparing the frequency of mutant emergence under *in vitro* and *in vivo* conditions. Because only A. baumannii experienced a dramatic shift in rifabutin MICs in RPMI versus MHII, we focused on two specific A. baumannii clinical isolates (HUMC1 and ABNIH1) for studies on the effect of medium on resistance emergence frequencies. In MHII, both isolates exhibited the same sensitivity to both rifampin and rifabutin (MIC = 3.13 mg/L). In RPMI, both isolates were equally susceptible to just rifampin (MIC = 3.13 mg/L). However, in RPMI, HUMC1 was highly susceptible to rifabutin (MIC ≤ 0.05 mg/L), while ABNIH1 was not (MIC = 3.13 mg/L). Serial passage allowed the selection of highly resistant mutants for both drugs in both isolates. Despite HUMC1 being more susceptible to rifabutin in RPMI media, it was still possible to select for resistant mutants (Fig. 4).

Using a high-inoculum plating strategy, the frequency of rifabutin resistance emergence was higher in RPMI (1E−05 to 1E−06) than MHII and tryptic soy agar (TSA) (1E−08 to 1E−10). The frequency of resistance emergence to both rifampin and rifabutin was similar when bacteria were cultured on the same media (Table 3) (Mann-Whitney, *P* > 0.05). However, the frequency of resistance emergence was higher when bacteria were cultured on RPMI agar than TSA for both rifampin (Kruskal-Wallis test; *P* = 0.014) and rifabutin (Kruskal-Wallis test; *P* = 0.028) (Table 3).

**TABLE 3:** *In vitro* and *in vivo* mutation frequencies^*a*^

Strain	Mutation frequency							
	TSA		MHII agar		RPMI agar		RPMI+AA agar	
	RFB (8 mg/L)	RIF (8 mg/L)	RFB (8 mg/L)	RIF (8 mg/L)	RFB (8 mg/L)	RIF (8 mg/L)	RFB (8 mg/L)	RIF (8 mg/L)
*In vitro*								
A. baumannii HUMC1	1.28E−08	1.44E−08	2.20E−09	9.19E−09	5.49E−06	9.89E−06	6.30E−07	1.71E−06
A. baumannii ABNIH1	5.95E−09	1.75E−08	8.85E−09	4.17E−08	1.40E−05	4.84E−05	6.83E−06	5.75E−06
A. baumannii ATCC 17978	8.49E−10	7.95E−09	5.72E−09	5.14E−09	2.12E−06	2.20E−05	7.57E−06	2.60E−05
A. baumannii LAC-4	7.41E−09	1.76E−08	1.29E−08	2.62E−08	1.79E−06	6.72E−06	1.06E−06	6.65E−06
S. aureus LAC	1.10E−07	9.49E−08	1.11E−07	1.08E−07	1.43E−06	8.60E−06	6.05E−06	3.09E−06
E. coli JJ1886	3.89E−08	2.21E−08	1.48E−07	1.95E−07	7.06E−07	1.06E−05	4.53E−07	5.70E−06

*In vivo*	Blood (TSA)		Kidney (TSA)		Blood (RPMI agar)		Kidney (RPMI agar)	
A. baumannii HUMC1	<1.7E−09	8.4E−08	<3.7E−08	1.2E−07	<8.3E−09	4.1E−07	3.7E−07	1.6E−06
A. baumannii LAC-4	<4.1E−09	<4.1E−09	<2.2E−09	<2.2E−09	<4.1E−09	<4.1E−09	<2.2E−09	<2.2E−09

a For matched medium conditions, there was no significant difference in the emergence frequency of resistant A. baumannii mutants (Mann-Whitney test; *P* > 0.05). However, there was a significant difference when the A. baumannii mutants selected on TSA and RPMI were compared for rifampin (Kruskal-Wallis, *P* = 0.014) and rifabutin (Kruskal-Wallis, *P* = 0.028). C3H mice (*n* = 3) were infected with 1.3E7 to 2.7E7 CFU of A. baumannii HUMC1 or 4.6E6 CFU of A. baumannii LAC4. Blood and kidneys were collected 16.5 h postinfection, and samples were plated on TSA or RPMI agar plates alone or supplemented with 8 mg/L rifampin or rifabutin.

### *In vivo* rifampin and rifabutin mutation frequency.

Because we found that the culture conditions affect A. baumannii susceptibility to rifabutin, as measured *in vitro* by MIC assay or frequency of mutant resistance emergence, we therefore sought to determine how exposure in an *in vivo* environment would affect the frequency of resistance emergence. We were unable to select for any rifampin- or rifabutin-resistant mutants when mice were infected and treated *in vivo* and blood or kidney homogenates were plated on antibiotic selective agar plates. The experiment was repeated, but mice were not treated with rifampin or rifabutin *in vivo* to allow harvesting of a greater number of bacteria in the blood and kidney homogenates. The mutation frequencies of HUMC1 collected from both blood and kidney were 0.5 to 2 log higher for rifampin than rifabutin on both RPMI agar and TSA. On the other hand, no difference in mutation emergence was observed under any of the conditions tested for LAC-4. This may be due to the generally lower mutation frequency observed in LAC-4 than HUMC1 (Table 3).

## DISCUSSION

We have shown that A. baumannii is significantly more susceptible to rifabutin when cultured in RPMI medium (9, 10). By screening a larger collection of A. baumannii, E. coli, S. aureus, K. pneumoniae, and P. aeruginosa clinical isolates, we demonstrated that hypersensitivity to rifabutin is commonly and uniquely observed in A. baumannii. Additionally, consistent with our previous study, we were unable to observe any hypersusceptible isolates in the non-Acinetobacter bacteria that were tested (9, 10).

We also found that under any medium condition, rifabutin resistance emergence rates were lower than the rates of rifampin resistance, which supports the potential clinical utility of adjunctive rifabutin for such infections (12). However, we observed a higher frequency of resistant mutants in RPMI agar selection plates than in MHII. Fortunately, the low frequency of resistant mutants *in vivo* was consistent with that observed *in vitro* on the nutrient-rich media such as MHII and TSA rather than on RPMI agar. The bacteria grow more slowly in RPMI, and this lower growth rate may affect the frequency of resistance emergence. Thus, paradoxically, RPMI is more accurate at predicting *in vivo* rifabutin efficacy but less accurate at predicting *in vivo* emergence of resistance to rifabutin for treatment of A. baumannii infection.

Consistent with other published studies, we were unable to select for any rifampin-resistant mutants in less than 48 h when mice were infected with A. baumannii and treated *in vivo* (13–15). However, A. baumannii rifampin-resistant isolates were reported to have been selected for in a murine pneumonia infection model between 48 and 72 h postinfection (16).

Based on *in vitro* susceptibility, *in vivo* treatment efficacy, and *in vitro* and *in vivo* frequency of resistance emergence, rifabutin remains a potential useful therapeutic for the treatment of A. baumannii infections.

## MATERIALS AND METHODS

### Serial passage mutant selection.

Two A. baumannii clinical isolates, HUMC1 and ABNIH1, were serially passaged for 20 days in broth supplemented with antibiotics as previously described (17). Briefly, bacteria were serially passaged 20 times with sub-MICs of rifabutin (R3530-25MG; Sigma) or rifampin (R3501-1G; Sigma) in MHII (90000-602; VWR) or RPMI (11875119; Gibco) media at 37°C and 200 rpm. Every day, 100 μL of culture was passaged into 10 mL fresh MHII or RPMI medium with antibiotics. During the first 5 days of passage, bacteria were grown in media with 1/3 the MIC of rifabutin or rifampin. Antibiotic susceptibility was tested by plating bacteria on TSA (211825; VWR) plates containing 3×, 10×, or 100× the passage concentration of antibiotic. The starting antibiotic concentration on day 6 was determined based on the agar plate result. The same testing process was performed on days 10, 15, and 20 to determine the starting antibiotic concentration of the subsequent 5 days.

### High-inoculum mutant selection.

There are no CLSI breakpoint interpretations for RIF or RFB against A. baumannii. We therefore referenced the RIF MIC breakpoint for staphylococci (4 mg/L). To increase the stringency of our conditions, we then then doubled that to reach the 8 mg/L that was used in our experiments. Bacteria were cultured overnight in tryptic soy broth (TSB) at 37°C and 200 rpm, and spontaneous rifampin- and rifabutin-resistant mutants were selected on TSA or MHII agar plates supplemented with 8 mg/L of rifampin or rifabutin. Alternatively, bacteria were cultured overnight in RPMI alone or RPMI plus amino acids (RPMI+AA) at 37°C and 200 rpm as previously described (9), and rifampin- and rifabutin-resistant mutants were selected on RPMI alone or RPMI+AA agar plates supplemented with 8 mg/L of rifampin or rifabutin.

### *In vivo* mutant selection.

Nine- to 15-week-old C3H mice (*n* = 3) were infected with 1.3E7 to 2.7E7 CFU of A. baumannii HUMC1 or 4.6E6 CFU of A. baumannii LAC-4 intravenously (18–20). Mice were euthanized at 16.5 h postinfection, and blood and kidney samples were collected. Kidneys were homogenized in phosphate-buffered saline (PBS). Serial dilutions were plated on drug selection plates to select for mutants and on nondrug plates to determine total CFU. Drug selection plates were supplemented with 8 mg/L rifampin or rifabutin in TSA or RPMI agar. Plates were incubated at 37°C overnight, and colonies were enumerated.

### MIC assay.

Unless otherwise indicated, the broth microdilution method was used to determine MICs (9, 21). The medium used for the MIC assays performed in the present study was either MHII alone or RPMI supplemented with 10% fetal bovine serum (PS-100; Phoenix Scientific). The final drug concentrations in the plate were 2-fold dilutions ranging from 0.05 to 25 mg/L. The inoculum concentration was confirmed by plating serial dilutions on TSA plates, incubating at 37°C overnight, and enumerating colonies. MIC plates were incubated at 35 ± 2°C without shaking, and results were recorded at the recommended time points (21).

### *In vivo* rifampin and rifabutin efficacy.

Galleria mellonella larvae (WAXB500; Timberline) were stored at room temperature in the dark for no longer than 1 week before experimentation. Larvae weighing 240 to 350 mg (group mean standard deviation ≤ 4.6) and grouped by tens were incubated at 4°C for up to 1 h before infection to reduce their movement during injection. Bacteria were prepared for infection by growing overnight in TSB at 37°C and 200 rpm and then subculturing in TSB until mid-log phase. Bacterial suspensions were washed 3 times with PBS and adjusted to an optical density (OD) of 0.5 before dilution to desired inocula. Each larva was disinfected by brief rolling in a 70% ethanol-soaked KimWipe (470224-038; Kimtech Science) and infected subcutaneously using an NE-1000 fully programmable single-syringe pump (New Era Pump Systems, Inc.) with 10 μL A. baumannii HUMC1, E. coli JJ1886, P. aeruginosa PAO1, K. pneumoniae KPC-KP1, or S. aureus LAC to determine respective 100% lethal doses (LD_100_). No-infection control larvae received 10 μL PBS. Larvae were then infected with previously determined LD_100_ (in CFU) of each strain and treated with 10 μL PBS or 1, 5, or 10 mg/kg rifampin or rifabutin 1 h postinfection. No-infection control larvae received 2 doses of 10 μL PBS. For all experiments, larvae were incubated in 100-mm petri dishes (25384-302; VWR) at 37°C, and survival was monitored up to day 4 postinfection. CFU were calculated by plating serial dilutions on TSA plates, incubating them at 37°C overnight, and enumerating colonies.
